# Brain and serum lipidomic profiles implicate Lands cycle acyl chain remodeling association with *APOEε4* and mild cognitive impairment

**DOI:** 10.3389/fnagi.2024.1419253

**Published:** 2024-06-11

**Authors:** Jason Mares, Ana Paula Costa, William J. Dartora, Krista M. Wartchow, Artur Lazarian, David A. Bennett, Tal Nuriel, Vilas Menon, Laura Beth J. McIntire

**Affiliations:** ^1^Center for Translational & Computational Neuroimmunology, Department of Neurology, Taub Institute for Research on Alzheimer’s Disease and the Aging Brain, Columbia University Irving Medical Center, New York, NY, United States; ^2^Lipidomics and Biomarker Discovery Lab, Department of Radiology, Brain Health Imaging Institute, Weill Cornell Medicine, New York, NY, United States; ^3^Rush Alzheimer’s Disease Center, Rush University Medical Center, Chicago, IL, United States; ^4^Department of Pathology and Cell Biology, Taub Institute for Research on Alzheimer’s Disease and the Aging Brain, Columbia University Irving Medical Center, New York, NY, United States

**Keywords:** lipidomics, lipid metabolism, Alzheimer’s disease, ROSMAP, ApoE

## Abstract

**Introduction:**

At least one-third of the identified risk alleles from Genome-Wide Association Studies (GWAS) of Alzheimer’s disease (AD) are involved in lipid metabolism, lipid transport, or direct lipid binding. In fact, a common genetic variant (ε4) in a cholesterol and phospholipid transporter, Apolipoprotein E (*APOEε4*), is the primary genetic risk factor for late-onset AD. In addition to genetic variants, lipidomic studies have reported severe metabolic dysregulation in human autopsy brain tissue, cerebrospinal fluid, blood, and multiple mouse models of AD.

**Methods:**

We aimed to identify an overarching metabolic pathway in lipid metabolism by integrating analyses of lipidomics and transcriptomics from the Religious Order Study and Rush Memory Aging Project (ROSMAP) using differential analysis and network correlation analysis.

**Results:**

Coordinated differences in lipids were found to be dysregulated in association with both mild cognitive impairment (MCI) and *APOEε4* carriers. Interestingly, these correlations were weakened when adjusting for education. Indeed, the cognitively non-impaired *APOEε4* carriers have higher education levels in the ROSMAP cohort, suggesting that this lipid signature may be associated with a resilience phenotype. Network correlation analysis identified multiple differential lipids within a single module that are substrates and products in the Lands Cycle for acyl chain remodeling. In addition, our analyses identified multiple genes in the Lands Cycle acyl chain remodeling pathway, which were associated with cognitive decline independent of amyloid-β (Aβ) load and tau tangle pathologies.

**Discussion:**

Our studies highlight the critical differences in acyl chain remodeling in brain tissue from *APOEε4* carriers and individual non-carriers with MCI. A coordinated lipid profile shift in dorsolateral prefrontal cortex from both *APOEε4* carriers and MCI suggests differences in lipid metabolism occur early in disease stage and highlights lipid homeostasis as a tractable target for early disease modifying intervention.

## Introduction

Dysregulation of the lipidome is strongly implicated in neurodegenerative diseases such as Alzheimer’s disease (AD) as well as during aging (Foley, 2010; Tu et al., 2017; Wong et al., 2017). In AD, lipid dysregulation AD is further implicated by genome-wide association studies (GWAS) in which one-third of risk variants are involved in lipid metabolism, lipid transport, or direct lipid binding (Kunkle et al., 2019). A cholesterol and phospholipid transporter, apolipoprotein E ε4 (*APOEε4*), is the strongest genetic risk factor for late-onset AD in individuals of European descent. Multiple other variants involved in phospholipid metabolism have been identified in GWAS studies associated with AD risk, including cholesterol transport protein Clusterin/ApoJ; lysophospholipid transporter ABCA7, membrane binding proteins TREM2 and SORL1 and a phosphoinositide phosphatase, INPP5D; Bridging integrator 1 (BIN1) and phosphatidylinositol binding clathrin assembly protein (PICALM) (Kunkle et al., 2019).

Additionally, lipidomic data confirm lipid dyshomeostasis associated with AD in autopsy brain (Batra et al., 2022), cerebrospinal fluid (CSF) (Dakterzada et al., 2023; Do et al., 2023), human plasma (Li et al., 2023), and animal models (Chan et al., 2012; Mcintire et al., 2012) pointing to the potential for specific pathway(s) in lipid metabolism to underlie multiple AD disease mechanisms. Specifically, the loss of polyunsaturated fatty acid (PUFA) includingdocosahexaenoic acid (DHA), across multiple lipid classes is regularly observed in these studies. In addition, aberrant uptake of DHA has been observed by age 30–35 in *APOEε4* carriers, who are at increased risk of developing AD (Yassine et al., 2017). Despite these multiple lines of evidence highlighting lipid dysregulation as a significant contributor to AD, a system-wide understanding able to synthesize these data has not yet been fully developed.

Based on the genetic and lipidomic evidence for phospholipid dysregulation leading to an increased risk of late-onset AD, we aimed to determine if transcriptomic and lipidomic analyses of the human brain support the hypothesis that dysregulation of phospholipid metabolism is associated with AD. We analyzed the existing lipidomics data from serum and the dorsolateral prefrontal cortex (DLPFC) autopsy tissue from the Religious Order Study and Rush Memory Aging Project (ROSMAP) cohort and identified multiple lipids that are dysregulated in association with Alzheimer’s disease progression. Our corresponding analysis of existing RNA-seq data from the ROSMAP and identified multiple genes in lipid metabolism associated with cognitive decline independently of the hallmark AD pathologies, amyloid-β (Aβ) load and tau tangle density.

Our analysis is the first, to our knowledge, to report coordinated lipid profile differences in the brain of both MCI and *APOEε4* carriers (NCI+), indicative of lipid dyshomeostasis early in disease progression. The dysregulated lipids and genes suggest that acyl chain remodeling deficits may be involved in the etiology and pathogenesis of cognitive impairment.

## Materials and methods

### ROSMAP cohort

The Religious Order Study (ROS) and Rush Memory and Aging Project (MAP) cohorts (Bennett et al., 2012a,b, 2018) are two prospective clinical-pathologic cohort studies of aging and dementia conducted by the Rush Alzheimer’s Disease Center. ROS started in 1994 with the recruitment of older individuals from Catholic religious communities across the United States. MAP started in 1997 with the recruitment of individuals from a wide range of backgrounds and socio-economic statuses from northeastern Illinois, United States. All participants are without known dementia at enrollment and agree to annual clinical evaluation and brain donation after death. Both studies were approved by an Institutional Review Board of Rush University Medical Center. Each participant signed an informed consent, Anatomic Gift Act, and repository consent allowing their data and biospecimens to be repurposed. After enrollment, participants are evaluated for cognitive and physical function annually, and diagnoses of dementia and it’s causes and MCI, and cognitive decline, as previously described (Bennett et al., 2002, 2006a,b; Oveisgharan et al., 2023). After death, a comprehensive pathologic assessment is performed for AD and other neurodegenerative and cerebrovascular pathologies, as previously described (Boyle et al., 2019, 2021). *APOE* genotype was determined as described (Yu et al., 2017). Here, lipidomics profiling data previously generated on 99 samples from the dorsolateral prefrontal cortex (DLPFC) brain region and 542 serum samples were used, with covariates including age at death (brain tissue), age of draw (serum), sex, post-mortem interval (brain tissue), apolipoprotein *E* ε4 (*APOEε4*) genotype status, education history, cognitive scores, clinical diagnosis at death, and Aβ (amyloid-β; load) and paired helical filament-tau protein (tangles) density in brain tissue. Demographic characteristics of the ROSMAP participants whose data were used in this study are included in Table 1.

**Table 1:** ROSMAP cohort overview.

BRAIN	NCI−	NCI+	MCI−	MCI+	AD−	AD+	*p*-value
Brain tissue (*N*, %)	41 (41.4)	6 (6.1)	22 (22.2)	6 (6.1)	15 (15.1)	9 (9.1)	
Age of death (Years, SD)	89.9 (5.7)	89.7 (6.2)	91.7 (6.3)	91.2 (5.4)	93.0 (6.4)	87.7 (5.8)	0.321
Sex, male (*N*, %)	13 (13.2)	3 (3.0)	7 (7.1)	0 (0)	3 (3.0)	0 (0)	0.138
Postmortem interval (hours, SD)	8.6 (9.0)	7.1 (2.0)	7.6 (2.8)	9.4 (4.1)	12.0 (8.5)	15.2 (3.0)	0.532
Years of education (mean, SD)	14.8 (3.4)	18.2 (3.8)	14.7 (2.7)	15.8 (1.6)	15.7 (2.4)	15.2 (4.7)	0.239
CGRS (mean, SD)	0.062 (0.042)	0.050 (0.032)	0.031 (0.051)	0.014 (0.061)	−0.048 (0.103)	−0.117 (0.100)	<0.001
Plaques (mean, SD)	0.549 (0.499)	0.568 (0.420)	0.784 (0.465)	0.980 (0.429)	0.920 (0.448)	1.484 (0.268)	<0.001
Tangles (mean, SD)	1.810 (0.639)	1.907 (0.791)	2.085 (1.089)	2.443 (1.626)	2.643 (1.265)	4.626 (1.277)	<0.001

SERUM	NCI−	NCI+	MCI−	MCI+	AD−	AD+	*p*-value
Serum (*N*, %)	356 (65.7)	70 (12.9)	81 (14.9)	22 (4.1)	9 (1.7)	4 (0.7)	
Age of draw (Years, SD)	81.3 (7.4)	80.8 (7.7)	87.2 (6.1)	85.3 (5.7)	89.5 (8.2)	78.5 (4.7)	<0.001
Sex—Male (*N*, %)	80 (14.8)	9 (1.7)	20 (3.7)	4 (0.7)	3 (0.6)	0 (0)	0.203
Years of education (mean, SD)	15.7 (3.1)	16.0 (3.0)	15.6 (3.1)	17.0 (3.5)	15.5 (2.6)	17.5 (6.2)	0.677
CGRS (mean, SD)	0.045 (0.053)	0.028 (0.078)	−0.010 (0.083)	−0.027 (0.094)	−0.027 (0.120)	−0.141 (0.100)	<0.001
Plaques (mean, SD)	0.593 (0.491)	0.666 (0.448)	0.954 (0.459)	1.375 (0.251)	0.885 (0.225)	1.758 (0.042)	<0.001
Tangles (mean, SD)	1.815 (0.967)	2.909 (1.960)	2.628 (1.280)	4.071 (1.341)	3.214 (1.309)	4.068 (0.397)	<0.001

RNA bulk	*APOEε4* non-carrier	*APOEε4* carrier	*p*-value
Brain tissue (*N*, %)	567 (74.1)	198 (25.9)	
Age of death (Years, SD)	89.4 (6.7)	88.5 (6.0)	0.004
Sex—Male (*N*, %)	193 (34)	71 (36)	<0.001
Postmortem interval (hours, SD)	8.1 (6.4)	7.3 (4.4)	0.004
Years of education (mean, SD)	16.4 (3.5)	17.0 (3.5)	0.005
RIN (mean, SD)	6.7 (1.4)	6.6 (1.35)	0.211
CGRS (mean, SD)	−0.00 (0.09)	−0.05 (0.11)	0.061
Plaques (mean, SD)	0.6 (0.51)	0.98 (0.48)	0.090
Tangles (mean, SD)	1.97 (1.12)	2.7 (1.45)	0.069

NCI, no cognitive impairment; MCI, mild cognitive impairment; AD, Alzheimer’s disease-associated dementia, and *APOEε4* carrier status (+/−); *N*, number of participants; SD, standard deviation; CGRS, cognitive global random slope of decline.

### Lipidomic data

For our analysis, we used previously generated lipidomic data generated using the Biocrates AbsoluteIDQ p180 platform (Biocrates AG, Innsbruck, Austria), and made available from the AMP-AD Knowledge Portal on Synapse at https://www.synapse.org/#!Synapse:syn26007829. The p180 platform is a multiplexed targeted metabolomic assay covering 188 metabolites in a variety of classes, including hexoses, amino acids, biogenic amines, acylcarnitines, glycerophospholipids, and sphingolipids (Arnold et al., 2020). Selective analyte detection was accomplished by use of a triple quadrupole tandem mass spectrometer operated in Multiple Reaction Monitoring (MRM) mode in which specific precursor to product ion transitions were measured for every analyte and stable isotope labeled internal standard. Data was generated by the Duke Metabolomics and Proteomics Shared Resource, a member of the Alzheimer’s Disease Metabolomics Consortium, using protocols published previously for blood samples. The platform has been validated for human plasma (Klavins et al., 2015; Trabado et al., 2017) and applied successfully to a variety of other matrices (St John-Williams et al., 2017; Varma et al., 2018; Weng et al., 2019). The Alzheimer’s Disease Metabolomics Consortium has previously published results from the p180 platform with the ROSMAP data (Arnold et al., 2020).

### RNAseq data

We used previously generated bulk RNA-sequencing data available from Synapse based on methods previously described (Mostafavi et al., 2018) and accessible on the AD Knowledge Portal at https://www.synapse.org/#!Synapse:syn3388564. Demographic information for the subset of participants which had RNA-sequencing data available is shown in Table 1.

### Data preprocessing

Lipid species with a high number of missing values (>17%) were removed and remaining missing values were imputed as zero because of the sensitivity limit of detection for the lipid panel. Since the majority of a lipid species is detected across the samples, those values that are missing may be interpreted as an undetectable level of lipid based on the limited sensitivity of the assay, and therefore represent a very low or 0 value of the lipid level instead of a missing value. Data was normalized by taking the mole percent of lipid species (relative abundance) within each sample and standardizing across lipid species. After normalization, we controlled for batch effects as well as sex, age, and post-mortem interval through the *lmfit* function in the *limma* R package. We performed additional supplementary analyses where the data was corrected for years of education during pre-processing or the model was adjusted for education where indicated. Otherwise, the data and analyses were not corrected/adjusted for education. For the brain and serum data, age at death and age at serum draw were used, respectively, as the age covariate. For serum data, we used a linear mixed model to control for repeated samples from the same donors. For all analyses using mixed models, donors with records of one-time visits were grouped together.

### Differential analysis of lipidomics

Sample label groupings: Each patient in the ROSMAP subset selected for analysis in this study had a clinical diagnosis falling into one of three categories: no cognitive impairment (NCI), mild cognitive impairment (MCI), or Alzheimer’s dementia (AD). In our analyses, these participants were further stratified into six individual groups, combining the subject’s clinical diagnosis and *APOEε4* carrier status (+/−). We treat NCI *APOEε4* non-carriers (NCI−) as the reference group throughout the analyses.

To assess lipid differences across disease progression, we performed *t*-tests of lipids species between NCI− and every other group to obtain a preliminary set of lipids with potential association with AD progression.

### Unsupervised learning

Correlation heatmaps were constructed to broadly compare the lipid profiles of diagnosis groups, using Spearman correlations between the lipid profiles of two diagnosis groups. More precisely, we derived the mean pairwise correlation of two groups by considering the average of all pairwise correlations between individual samples of one group with those of the latter group. We then adapted the weighted gene co-expression network analysis (WGCNA) approach adapted for lipid species data. WGCNA performs hierarchical clustering on the (dis)similarity matrix derived from the data’s topology to produce modules (Langfelder and Horvath 2008). Here, we derived “eigenlipids” for each module by calculating the first PC using only the lipids assigned to the module of interest. For each module, we performed Mann–Whitney tests pairwise across all six diagnosis-genotype groups. We opted for this non-parametric test to account for outliers and skewed distributions of eigenlipids of lipid modules.

For the *K*-means clustering and module analyses, we incorporated lipids with at least one statistically significant (*p* < 0.05) difference between the NCI− group and any other diagnosis-carrier status group via *t*-test. In the *K*-means clustering analysis, we used Euclidean distances for brain and serum sample data. The hyperparameter *k* for each analysis was selected after reviewing scree, elbow, and silhouette plots with *k* ranging from 2 to 10.

### Supervised learning

A set of linear models was used to examine the associations between lipid species and the outcome variable of cognitive global random slope, which is the estimated person-specific rate of change in the global cognition variable over time (i.e., cogng_demog_slope). We also analyzed the cognitive global random slope measure controlled for demographics and pathology (i.e., cogng_path_slope). Within these models, we controlled for age at death, education, post-mortem interval, and sex. Models only include samples with complete data. Benjamini–Hochberg (BH) correction was used to adjust the *p*-values for multiple comparisons.

### Mediation analysis of education

Mediation analysis was performed to investigate the potential mediating effects of education on a module produced from WGCNA of the brain data. The models involved an identified module from the brain data (*independent variable*), education (*potential mediator*), along with age, sex, and post-mortem interval (*control variables*) to predict between NCI−/NCI+ and NCI−/MCI− (*outcomes*). We used the R package *pysch* to conduct this series of mediation analyses.

## Results

### Summary statistics of datasets

We analyzed the lipid content of 99 post-mortem brain samples derived from ROSMAP participants, including 26 (26.3%) males and 21 (21.2%) *APOEε4*-carriers, with a mean age at death of 90.6 (±6.0) years old; 24 (24.2%) were diagnosed with AD at the time of death, 28 (28.3%) had MCI, and 47 (47.5%) had NCI (Table 1). This data is derived from a panel of 119 lipid species in the dorsolateral prefrontal cortex (DLPFC) brain region. We compared these findings to the analogous dataset of lipid content in serum samples from the same ROSMAP subjects, including 116 (21.5%) males and 96 (17.8%) *APOEε4* carriers, with a mean age of 83.8 (±6.6) years old (Table 1). Out of the 542 samples from the serum data, 13 (2.4%) were linked to individuals with AD, 103 (19.0%) with MCI, and 426 (78.6%) with NCI. Serum lipidomics included 125 lipid species from five lipid classes: acylcarnitines (C), diacyl-phosphatidylcholines (PC.aa), acyl-alkyl-phosphatidylcholines (PC.ae), sphingomyelins (SM), lysophosphatidylcholines (lysoPC) after quality control and filtering. Of the 111 subjects whose brain tissue was profiled using the Biocrates p180 platform lipid panel, we analyzed those with no other factors contributing to impairment, which resulted in 99 subjects (Table 1). Of these 99 brain tissues, 60 of them have accompanied RNA sequencing data. For the bulk RNA sequencing data, a total number of 765 brain tissue samples were analyzed, 264 (34.5%) of which were from males (Table 1). We compared the demographic and pathological data stratified by *APOEε4* carrier status of the subjects with RNA sequencing data in Table 1.

### Lipidomic differences across disease progression

**Figure 1:**
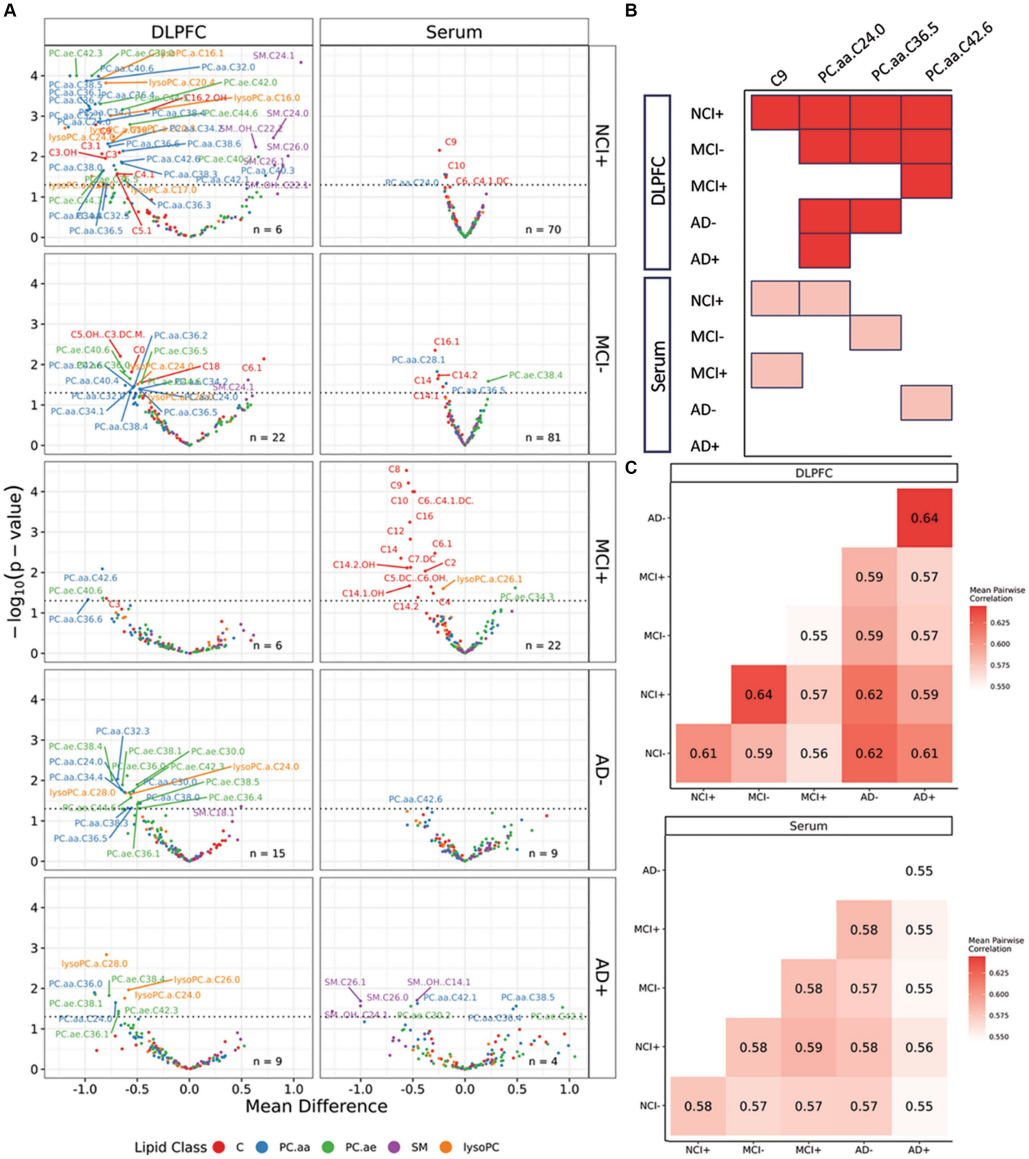
Differential lipids across dorsolateral prefrontal cortex and serum samples. **(A)** Volcano plots from *t*-tests of lipid species normalized mole percent between NCI− individuals and other diagnosis-carrier groups, where +/− indicates *APOEε4* carrier status. Left column, brain samples. Right column, serum samples. Dashed horizontal line indicates a threshold of *p*-value: 0.05. Lipid species are grouped by five lipid classes: acylcarnitines **(C)**, diacyl-phosphatidylcholines (PC.aa), acyl-alkyl-phosphatidylcholines (PC.ae), sphingomyelins (SM), lysophosphatidylcholines (lysoPC). **(B)** Lipids downregulated in both brain and serum across all six diagnosis-carrier groups. **(C)** Mean values for pairwise correlations at the individual level across all six diagnosis-carrier groups in brain and serum data, shaded by level of correlation.

To assess lipid differences across disease progression, we combined clinical diagnosis (NCI, MCI, and AD) with the *APOE* genotype, stratified by presence of the ε4 allele (+), resulting in 6 groups (NCI ε4+, NCI ε4−, MCI ε4+, MCI ε4−, AD ε4+, AD ε4−). From the normalized brain data, we performed *t*-tests between NCI− and every other group to identify candidate lipids with association with *APOEε4* carrier status and cognitive impairment. There were 67 lipid species with statistically significant differences (*p* < 0.05) between the NCI *APOEε4* non-carriers (NCI−) and at least one other group, and 32 lipids that were found to be significantly different in the serum data (Figure 1A). We observed that most of these differential lipids are downregulated in other groups relative to the NCI− group, suggesting a loss of function. Only four lipids were downregulated in both the brain and serum: these included C9, which was downregulated in brain and serum of NCI+, and serum of MCI+; PC.aa.C24.0, which was downregulated in brain and serum of NCI+, and brain of MCI−, AD− and AD+; PC.aa.C36.5, which was downregulated in brain and serum of MCI−, and brain of NCI+ and AD−; and PC.aa.C42.6, which was downregulated in serum of AD−, and brain of MCI−, MCI+ and NCI+ (Figure 1B). However, there was no overlap between brain and serum lipids upregulated relative to NCI *APOEε4* non-carriers (NCI−).

By correlating differential lipids across the six diagnosis-carrier status groups, we observed the strongest mean pairwise Spearman correlation between the NCI *APOEε4* carriers (NCI+) and MCI *APOEε4* non-carriers (MCI−) groups (*r_s_* = 0.64) in the brain, indicating a similar lipid profile shift between *APOEε4* carriers and MCI non-carriers (Figure 1C). This suggests that dysregulation of brain lipid profiles may occur early in the disease process, proposing a potential role in its etiology or pathogenesis. We also observed a high correlation between lipid profiles from brain of AD *APOEε4* non-carriers (AD−) and AD *APOEε4* carriers (AD+) (*r_s_* = 0.64), indicating that at late disease state, the *APOEε4* carriership has a smaller effect on differential lipids in affected and non-affected brain. These results highlight the similarity in lipid profile between healthy *APOEε4* carriers and MCI non-carriers in the lipid profiles within the brain, suggesting a profile shift in early disease progression similar to the chronic lipid dysregulation harbored by *APOEε4* carriers.

Whereas individual lipids show differential abundance across groups, aggregating lipids into modules allows for identification of groups of lipids that are co-regulated, suggesting biological relevance to lipid metabolic pathways. To this end, we performed module analysis with weighted lipid correlation network analysis (WLCNA) and opted to use the differential lipids from Figure 1 to enrich lipids that are significantly different across disease groups. We examined broad patterns of these differential lipids with a significant ANalysis Of VAriance (ANOVA) result across diagnosis-carrier status groups in the serum data [as mole percent (Mol%)] (Supplementary Figure S1) and used WLCNA (with L standing for lipids, instead of genes) to perform hierarchical clustering and identify modules from the correlation matrix derived across all the samples. For each module, we derived a module eigenlipid defined as the first principal component of the expression matrix of all lipids in the module. The module eigenlipid encapsulates the largest axis of variation and presents a reduced one-dimensional summary of the lipid abundance profile for that module. Importantly, we used “unsigned” modules, which include lipids with strong correlation or anti-correlation across samples, meaning that the overall sign of the eigenlipid is arbitrary and does not reflect the direction of the change in lipid abundance.

**Figure 2:**
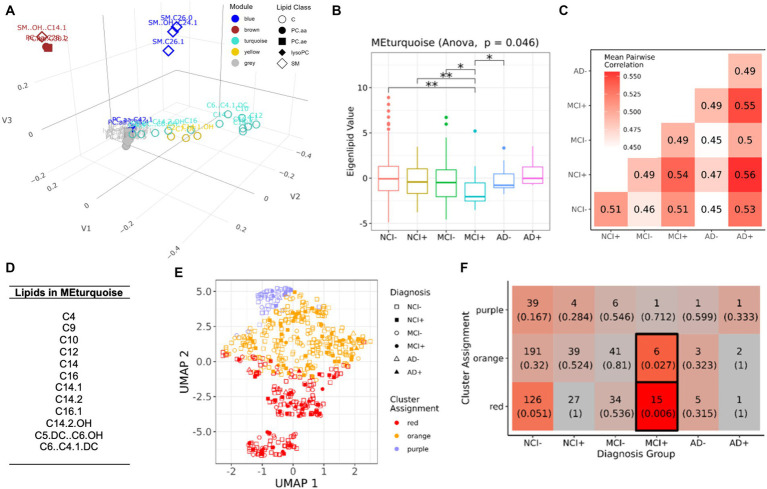
Module analysis of lipidomics in serum. **(A)** 3D projection of lipid species in the first three MDS dimensions, module assignment for each lipid species is indicated by color. **(B)** Comparison of the first eigenvalues for the turquoise module across diagnosis-genotype groups. Post-hoc pairwise testing is conducted for modules with significant results: *p < 0.10, **p < 0.05, and ***p < 0.01. **(C)** Correlation patterns of lipid profile in turquoise module across the six diagnosis-carrier status groups. **(D)** A list of lipid species in the turquoise module. **(E)** UMAP representation (*x* and *y*-axis) of turquoise module, samples are grouped using *k*-means clustering (*k* = 3). Samples are marked by cluster assignment (color) and diagnosis-genotype (shape). *APOEε4* carriers are marked by a filled-in bubble. **(F)** Heatmap table of *k*-means cluster assignments and diagnosis-genotype groups with outlined boxes and intense red shades indicating significant Fisher’s exact test result when considering marginal tables for each cell (*p* < 0.05).

We found five modules in serum (Figure 2; Supplementary Figure S2), of which the turquoise module (Figure 2A) shows significant differences among the six diagnostic groups stratified by *APOEε4* status (ANOVA, *p* = 0.024). Post-hoc pairwise testing of the turquoise module eigenlipid shows that it is differential in *APOEε4* carriers with MCI (MCI+) compared to all the other groups except *APOEε4* carriers with AD (AD+) (Figure 2B). Further, pairwise correlation of only lipids from the turquoise module shows little similarity (*r_s_* = 0.49–0.56) across disease diagnosis or *APOEε4* carrier status (Figure 2C), suggesting MCI+ individuals harbor a lipid profile in serum that is distinct from the other groups. Interestingly, the turquoise module consists solely of acylcarnitine species (Figure 2D), which is consistent when adjusting for education in the model (Supplementary Figure S3).

To determine if patterns across lipid species in the turquoise module are shared between samples, we applied *K*-means clustering of the samples in the serum data. After determining an optimal number of clusters (*k* = 3) (Figures 2E,F), we found that MCI+ samples were enriched in the red and orange clusters (Fisher’s exact test, Figure 2F), but no other diagnostic group showed enrichment in any cluster, suggesting that serum lipid profile biomarkers, including acylcarnitine species, may only be applicable to MCI *APOEε4* carriers (MCI+) and not for other diagnostic groups.

**Figure 3:**
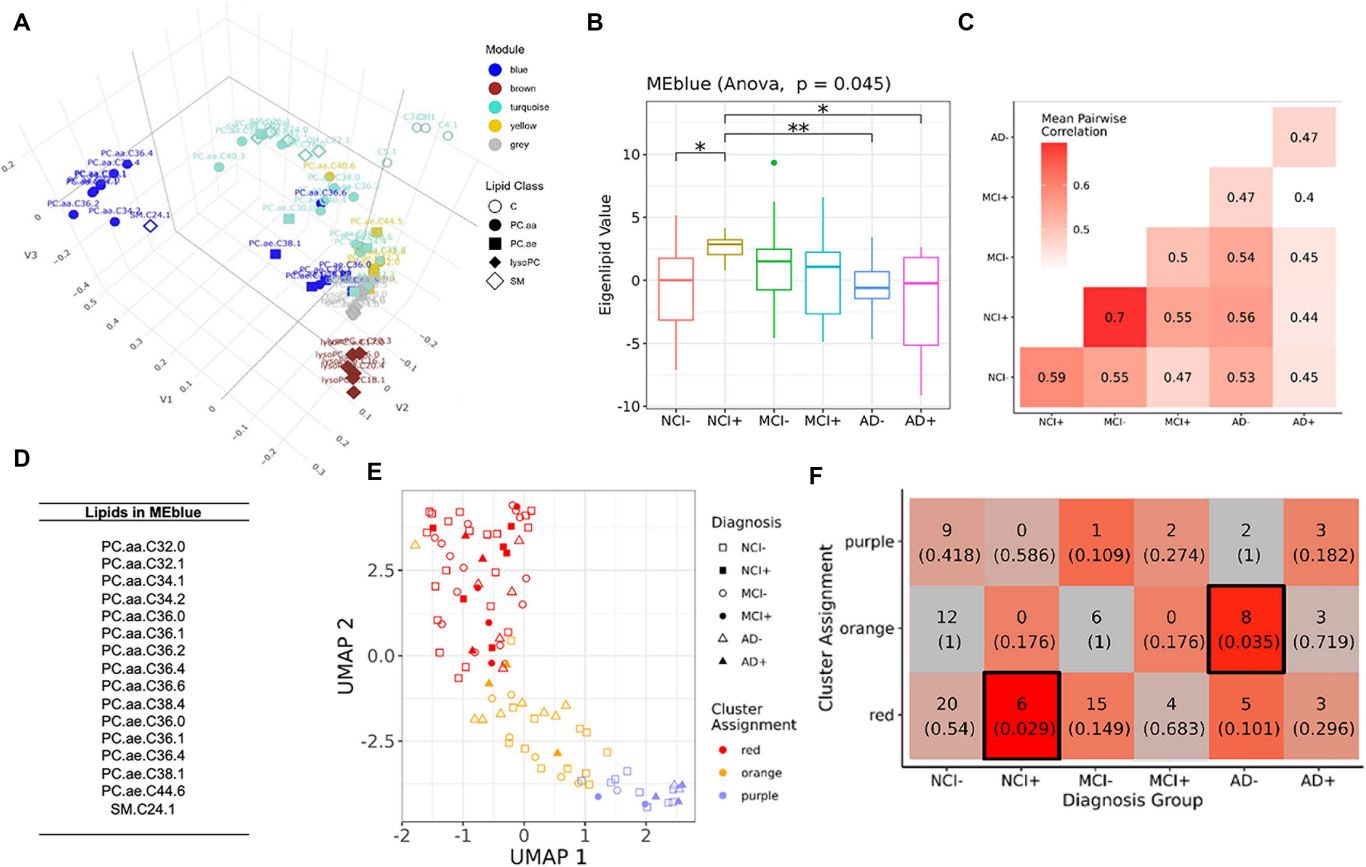
Module analysis of lipidomics in dorsolateral prefrontal cortex. **(A)** 3D projection of lipid species in the first three MDS dimensions, module assignment for each lipid species is indicated by color. **(B)** Comparison of the first eigenvalues for the blue modules across diagnosis-genotype groups. Post-hoc pairwise testing is conducted for modules with significant results: *p < 0.10, **p < 0.05, and ***p < 0.01. **(C)** Correlation patterns of lipid profile in blue module across the six diagnosis-carrier status groups. **(D)** A list of lipid species in the blue module. **(E)** UMAP representation (*x* and *y*-axis) of lipids in blue module, samples are grouped using *k*-means clustering (*k* = 3). Samples are marked by cluster assignment (color) and diagnosis-genotype (shape). *APOEε4* carriers are marked by a filled-in bubble. **(F)** Heatmap table of *k*-means cluster assignments and diagnosis-genotype groups with outlined boxes and intense red shades indicating significant Fisher’s exact test result when considering marginal tables for each cell (*p* < 0.05).

To determine if differential lipids in serum reflected coordinated changes in the brain, we next analyzed lipidomics from brain samples using a similar analytical pipeline as used for serum lipidomic data (Figure 3; Supplementary Figure S4). We found no overlapping species between brain and serum which were upregulated between NCI *APOEε4* non-carriers (NCI−) and any other group (Figure 1A). However, four lipids were downregulated in brain and serum (C9, PC.aa.C24.0, PC.aa.C36.5, and PC.aa.C42.6, but not in the same diagnostic and genotypic groups). We next performed module analysis to determine whether groups of lipids co-varied by diagnostic and genetic categories in the brain tissue data. We identified five modules (Figure 3A), of which the blue module showed significantly differential eigenlipid variation across the diagnostic-*APOEε4* carrier groups (Figure 3B; Supplementary Figure S4, ANOVA *p* = 0.045). Post-hoc pairwise testing of the blue module eigenlipid showed that all NCI+ and MCI− were different from other diagnostic groups but not from each other, suggesting a similar lipid profile shift in these two disease states (Figure 3B). The distributions of the eigenlipids for the remaining modules are shown in Supplementary Figure S4. In contrast to the blue module, the other module eigenlipids do not show differential profiles across the donor groups (Supplementary Figure S4).

Correlation analysis of the diagnosis-carrier status groups subsetting for blue module lipids from the brain showed highest similarities between NCI+ and MCI− (*r_s_* = 0.7, Figure 3C), a stronger signal compared to that seen in the serum in Figure 1B. Most of the lipids in the blue module are polyunsaturated phosphatidylcholines (PC), which are lower in the NCI+ group, suggesting dysregulation of a specific metabolic pathway involving activation of phospholipase A2 (PLA2) (Figure 3D). The brown module was enriched for lysoPC species (Supplementary Figures S5, S6), which is the product of PLA2, and is therefore expected to be higher with greater PLA2 activity. However, differential analysis of the lysoPC species in this module showed that lysoPC species are downregulated in early AD, contrasting with the expected greater lysoPC production as a result of higher PLA2 activity (Supplementary Figure S6), suggesting dysregulation of a pathway in lipid metabolism consistent with acyl chain remodeling.

Because education levels have been reported to moderate the effects of AD pathology on cognitive function in this cohort (Bennett et al., 2003), we next performed a correlation analysis of the lipids in the blue module in the brain data correcting for education at the pre-processing step to factor in the role of educational attainment in the lipidomics profiles, especially in the context that NCI+ samples are associated with higher years of education compared to the rest of the cohort. We observe that the correlation between NCI+ and MCI− no longer has the highest magnitude correlation; however, the correlations between NCI+ with MCI+ and AD+ are evident as cold spots.

*K*-means clustering of brain samples using blue module lipids yielded an optimal number of 3 clusters (Figures 3E,F). Interestingly, NCI+ and MCI− follow the same general trend (Figure 3E), suggesting similar differences in lipid profiles between these two groups; although only the NCI+ category showed statistically significant enrichment in the red cluster (Figure 3F), the majority of MCI− donors are also found in this cluster, supporting the similarity between NCI *APOEε4* carriers and MCI non-carriers.

**Figure 4:**
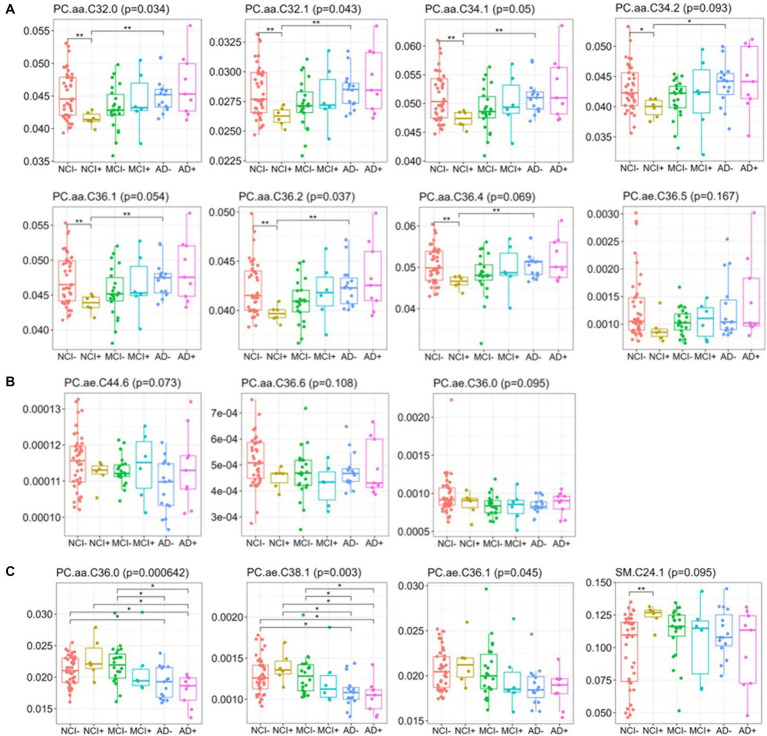
Relative abundance of blue module lipids across diagnosis-carrier groups in dorsolateral prefrontal cortex data. Box-and-whisker plots for lipids across the six diagnosis-carrier groups and post-hoc tests between NCI− and all other groups in the brain data.

Importantly, differences in lipids from the blue module (mole percent) are similar between NCI+ and MCI− (Figure 4). From the DLPFC, mole percent quantification of lipids in the blue module identified three distinct, biphasic patterns across disease progression (Figure 4). The patterns consist of (1) lipids that are lower in *APOEε4* carriers and MCI and greater across disease severity including PC.aa.C32.0; PC.aa.c32.1; PC.aa.C34.1;; PC.aa.C36.1; PC.aa.C34.2, PC.aa.C36.2; PC.aa.C36.4; PC.ae.C36.5 (Figure 4A); (2) lipids that trend toward being lower in all other groups compared to control including PC.ae.C36.0, PC.aa.C36.6; PC.ae.C44.6, and PC.aa.C36.6 (Figure 4B) or (3) those that trend to be higher in *APOEε4* carriers and MCI and decrease across disease severity including PC.aa.C36.0; PC.ae.C36.1; PC.ae.C38.1; SM.C24.1 (Figure 4C). This suggests that the lipid profile associated with *APOEε4* carriership is similar to the lipid profile associated with MCI, but dissimilar from other disease states and also highlights the non-linearity of the lipid profile shift across disease progression. Our results also indicate lipid species specific differences across disease progression within each lipid class.

We also considered whether adjusting for education during pre-processing would affect the modules in serum and DLPC brain tissue (Figures 2, 3). After running module analysis for the education-corrected serum data, only one module (turquoise) is identified that significantly differs across groups (Supplementary Figure S3). The turquoise module in Figure 2 is similar to the turquoise module in this analysis from the education-corrected serum data, with six lipids being shared between these two modules including C10, C12, C14, C14.1, C14.2, and C16.1. This new turquoise module maintains a significant differentiation between MCI+ and non-cognitively impaired carriers and non-carriers (NCI− and NCI+) (*p* < 0.05) (Supplementary Figure S3B). However, the new turquoise module corrected for education, displayed hotspots in the correlation heatmaps from MCI+/AD− and NCI+/AD− (Supplementary Figure S3C) in contrast to the previously identified correlations between MCI+/AD+. Using *k*-means clustering of serum samples from lipid species in the new turquoise module, we identified two clusters, though neither is significantly enriched in a specific cohort, though the cluster assignment for MCI carriers (MCI+) approaches significance (*p* = 0.06) (Supplementary Figure S3F) consistent with the previous analysis not corrected for education (Figure 2F).

To determine whether education is associated with the lipid modules, we also corrected the lipidomics dataset from DLPC brain for education at pre-processing. We identified a new magenta module (Supplementary Figure S7) that corresponds to the blue module from the initial analysis (Figure 3). The number of lipid species assigned to the new magenta module nearly doubles that of the initial blue module for the DLPC brain tissue. Interestingly, most of the lipid species from the blue module are also found in the new magenta module. The distributions of the eigenlipids of the magenta module are significantly different between non-impaired APOEe4 carriers (NCI+)—and non-carriers (NCI−), however, NCI+ and MCI− similarities are no longer present (Supplementary Figure S7). Cluster analysis of the lipid species in the new magenta module also showed a significant depletion of NCI+ samples in the new red cluster and a trend toward a depletion of NCI− samples in the new purple cluster suggesting differentiation of the NCI groups from all other diagnostic groups.

We found that the brain lipid profile in *APOEε4* carriers was correlated with MCI only when education was not included as a covariate. When education was included as a covariate, the correlation was reduced (Supplementary Figure S7). The *APOEε4* carriers in this study who are not cognitively impaired (NCI+) have a strong trend toward a higher education level overall and the age of death was not significantly different from non-carriers (Table 1), suggesting that the NCI+ group may be considered resilient in this study. Since we found a similar lipid profile in the resilient *APOEε4* carriers and MCI, this suggests that the lipid profile found in both *APOEε4* carriers (NCI+) and MCI non-carriers (MCI−) may be compensatory.

**Figure 5:**
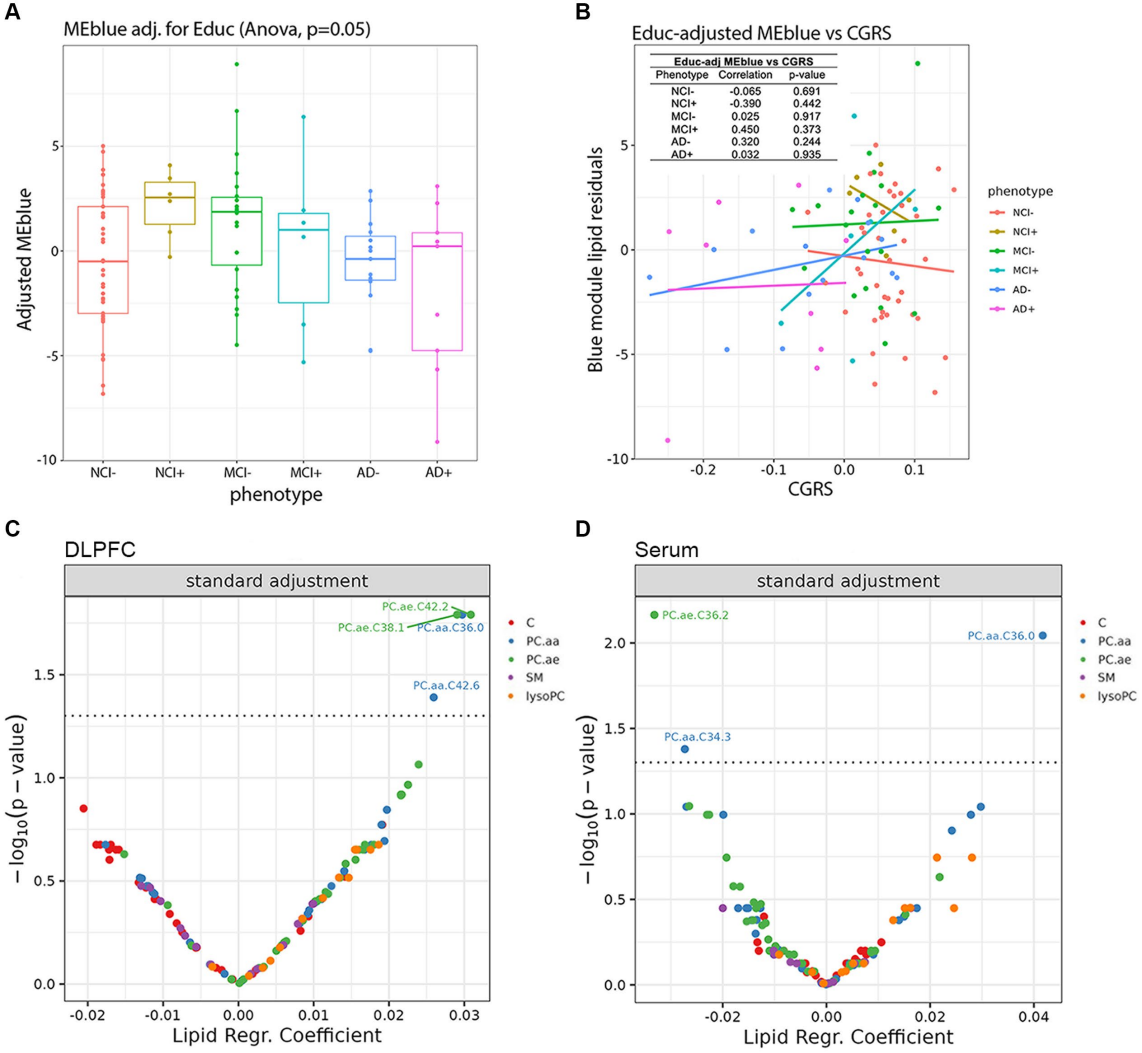
Impact of lipid species in the dorsolateral prefrontal cortex and serum on cognition controlling for educational background and neuropathological markers. **(A)** Education-adjusted scores for the blue eigen module are shown in diagnosis-genotype groups. **(B)** Association of the residuals of education-adjusted blue modules with cognitive global random slope are shown and post-hoc linear regression lines are added onto the plot. The left-top table reports correlation test results between residuals and cognitive global random slope for each diagnosis-genotype group. **(C,D)** Using linear models, we analyzed the association of the cognitive global random slope with each lipid species in the **(C)** DLPFC and **(D)** serum data. Volcano plots present the main results of these models with lipid species beta estimates and Benjamini-Hochberg-adjusted *p*-values shown along the *x* and *y* axes, respectively and significant results are shown above the threshold (dashed line).

We next considered the relationship between the brain lipids in the blue module found in Figure 3 and global cognition. After correcting for education in the blue module at the pre-processing stage, we found that education does not make a significant impact in the *APOEε4* carriers, as the previously observed difference between NCI− and NCI+ remains (Figure 5A). Using education-corrected blue module lipids, we plotted this measure against cognitive global random slope and show correlation measures and significance values (Figure 5B). Further, the trend for the similarity between NCI+ and MCI− remains. Interestingly, carriers and non-carriers with the least cognitive impairment have a high blue ME profile which is precipitously lost during the MCI+ stage and absent in AD+ (Figure 5B).

To examine the potential mediating role of education in the relationship between the blue module lipids and genotype/phenotype in brain, we conducted a series of mediation analyses. We considered the binary variable of non-cognitively impaired *APOEε4* non-carriers (NCI−) compared to non-cognitively impaired *APOEε4* carriers (NCI+) and NCI− compared to mild cognitive impairment *APOEε4* non-carrier (MCI−) as the outcomes for the model. The model did not find a significant association between the direct effect between the blue module lipids and NCI−/NCI+ status (*β* = 0.04, *p* = 0.024); integrating the education variable into the mediation model, this effect remained non-significant (*β* = 0.03, *p* = 0.062). The mediating effect of education also was non-significant (*β* = 0.26 *p* = 0.104). A similar non-significant result on the mediating role of education was found in the analysis between NCI− and MCI− (*β* = 0.15, *p* = 0.218); however, the direct effect between the blue module and NCI− versus MCI− was significant (*β* = 0.04, *p* = 0.037). Therefore, our findings suggest that within the examined population, education does not serve as a mediating factor in the association between the blue module and phenotypic outcomes in the comparisons of NCI−/NCI+ and NCI−/MCI−. This implies that the observed relationships between the blue module and phenotypes can be attributed to direct effects rather than mediated through educational attainment in the sample.

We next used supervised learning approaches to examine the relationship between lipid species abundance in the brain and global cognition, as shown in Figure 5. We used the random slope of cognitive measurement, which is not adjusted for pathology. In the first set of regression models (Figure 5C), we controlled for demographic variables, including age of death, sex, and education. We observe four lipids significantly associated with cognitive decline: PC.aa.C36.0, PC.aa.C42.6, PC.ae.C38.1, and PC.ae.C42.2 (Figure 5C), and three lipids in serum: PC.aa.C34.3, PC.aa.C36.0, and PC.ae.C36.2 (Figure 5D). We also ran the same model, except we used a pathological measure-adjusted version of cognitive global random slope; however, no lipids remained significant after correction for multiple comparisons (data not shown). All the nominally significant phosphatidylcholines in the brain show a positive association with cognitive global random slope indicating worsening with a decrease in the lipid level. This is consistent in the differential analysis of two of these lipids found in the blue module, PC.aa.C36.0 and PC.ae.C38.1 late in disease state (AD− and AD+) (Figure 4).

**Figure 6:**
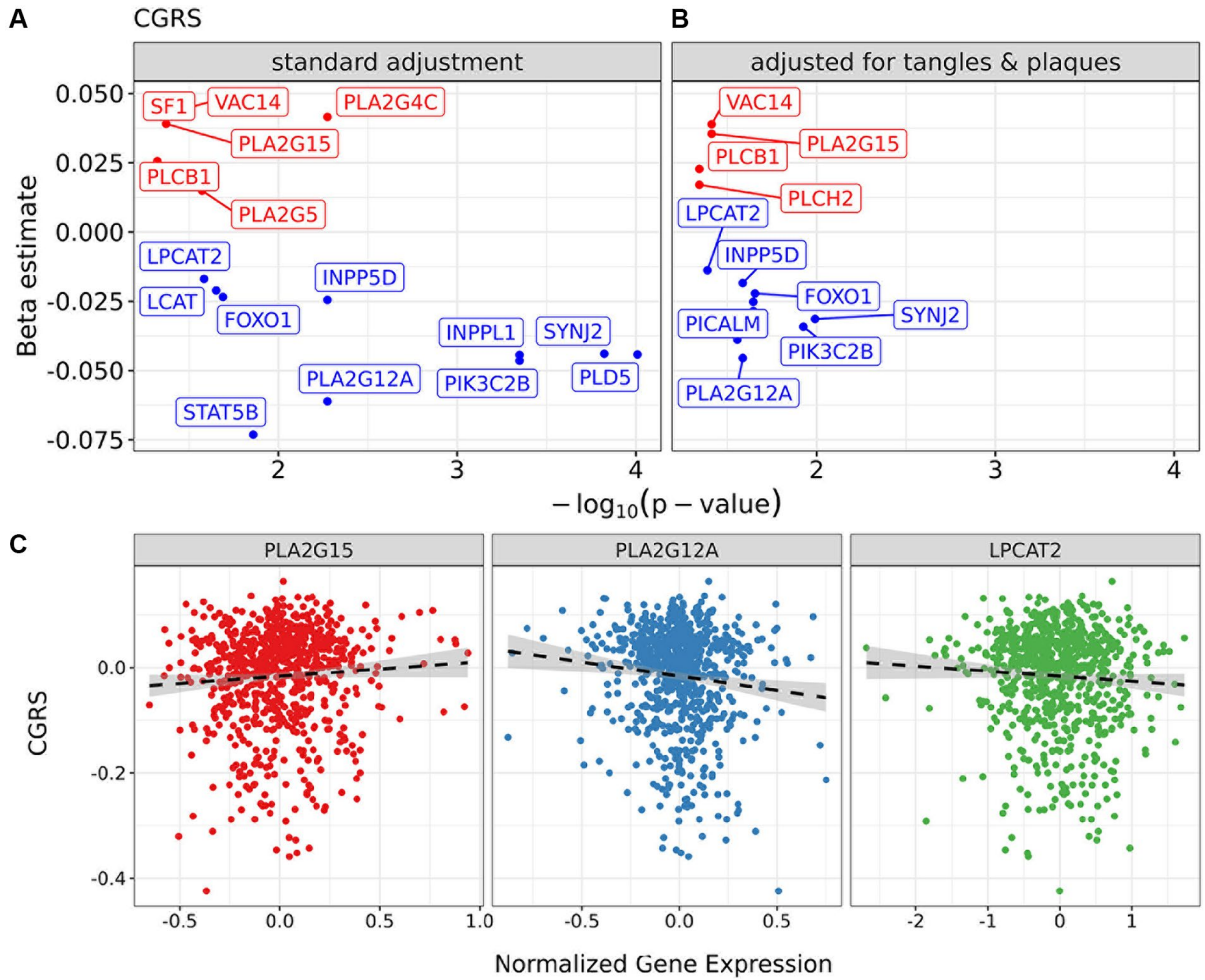
Impact of candidate genes in lipid metabolism in the dorsolateral prefrontal cortex on cognition controlling for neuropathology. Regression models for cognitive global random slope in bulk-RNA seq data with a focus on selected markers. **(A)** Volcano plots present results of regression models adjusting for sex, RIN, and age of death. **(B)** The panel shows the model also controlling for tangles and plaques. **(C)** Scatter plots of normalized gene expression for markers PLA2G15 and PLA2G12A and cognitive global random slope values in samples. A post-hoc regression line is added, distinct from those in **(A)**, to emphasize the direction of association between gene marker and clinical measure.

To contextualize the differences in lipid metabolism, we examined the expression of genes that are part of corresponding lipid metabolic pathways (Supplementary Figure S8A), and their putative associations with cognitive decline. We selected genes involved in lipid metabolism from a candidate panel identified in relation to phosphatidylcholine metabolism, which was the major class identified in brain in this study (Figures 3D, 4; Supplementary Figure S6). We found that candidate gene expression from post-mortem dorsolateral prefrontal cortex tissue was associated with the slope of cognitive decline when adjusting for technical batch effects, age of death, RNA integrity number (RIN), education and sex (Figure 6A). Interestingly, when controlling for Aβ load and tau tangle density as additional covariates, the candidate genes retained their associations, suggesting genetic drivers in lipid metabolism with effects on cognitive decline that are independent of these two classic AD pathologies (Figure 6; Supplementary Figure S8). Phospholipase A2 isoforms *PLA2G15* and *PLA2G12A* are two of the genes with the strongest association between expression and cognitive slope (Figure 6B). Though the isoform specific contributions to Lands cycle are not known, multiple PLA isoforms exist with lipid species and cell type specificity (Yarla et al., 2016; Murakami et al., 2017, 2020). Dysregulated expression of these genes suggests potential for regional and/or cell-specific regulation of PLA2 activity leading to changes in lipid metabolism which cannot be resolved using bulk lipidomic studies and may benefit from emerging spatial imaging mass spectrometry based lipidomics. Further, lysoPC acyl transferase2 (*LPCAT2*) shows a negative association with cognitive slope, indicating worsening with a higher level of expression, consistent with loss of lysoPC species in NCI+ and MCI− in differential analysis (Supplementary Figure S4) and in the lysoPC from the brown module (Supplementary Figures S6A,B).

## Discussion

The results from the present study support that the differences in serum and brain lipidomics implicate acyl chain remodeling is associated with *APOEε4* genotype and disease stage. A distinct lipid profile was observed in DLPC brain tissue from NCI *APOEε4* carriers (NCI+), which was also observed in MCI *APOEε4* non-carriers (MCI−) but not in NCI *APOEε4* non-carriers (NCI−) (Figures 3, 4). This suggests a stereotypical dysregulated lipid profile shift associated with disease risk in *APOEε4* carriers prior to symptomatic onset, similar to the lipid profile shift observed in MCI *APOEε4* non-carriers. These findings suggest that lipid dyshomeostasis may be most prominent early in disease etiology and that *APOEε4* contributes to a similar shift in the lipid profile. The defining lipids in the identified blue module (Figure 3D) and turquoise module, when adjusting for education (Supplementary Figure S7), are enriched in phosphatidylcholine species, including highly unsaturated lipid species with 4–6 double bonds, indicating that dyshomeostasis of polyunsaturated PC species may be driving early changes in the brain lipidome.

Given that a majority of the phosphatidylcholine species were downregulated in NCI *APOEε4* carriers (NCI+) and MCI non-carriers (MCI−), this suggests a greater phospholipase A2 (PLA2) activity, which has been previously reported in association with AD and in mouse models (Sanchez-Mejia et al., 2008; Sanchez-Mejia and Mucke, 2010). However, we did not observe greater lysoPC species, the product of PLA2 activity, which we identified in the brown module and differential analysis (Figure 3; Supplementary Figure S6) suggesting more complex regulation than an isolated effect on PLA2 activity. These differences in brain lipids can be modeled by dysfunction of the acyl chain remodeling pathway, which is referred to as Lands cycle (Lands, 1958; O’donnell, 2022). This dysregulation suggests more complex cellular regulation of PC and LPC than a single enzymatic reaction mediated by PLA2. The result is consistent with stimulation of acyl chain remodeling which would utilize excess LPC in the remodeling reaction mediated by lysophosphatidylcholine acyl transferases (LPCATs) in the Lands cycle (Supplementary Figure S9). Supporting this hypothesis, we observed the dysregulation of two isoforms of PLA2 and LPCAT2, which are in the Lands cycle pathway, however the isoform specific contributions are not yet known (Yarla et al., 2016; Murakami et al., 2017, 2020) (Figure 6). In fact, biphasic and age-dependent disruption of acyl chain remodeling has recently been reported in two mouse models of AD (Granger et al., 2019). Further, phospholipid dysregulation has been reported in mouse models overexpressing *APOEε4* by insertion of the human *APOEε4* allele in the mouse *APOE* locus (Zhu et al., 2015). While previous studies have reported an association between lipid metabolic pathways and AD, our work provides evidence for dysregulation in a specific lipid metabolic pathway and identifies molecular drivers that may lead to progressive differences, particularly implicating Lands cycle in association with *APOEε4* genotype and disease progression.

Comparing differential lipids from serum and brain tissue (Figure 1), our findings are consistent with those reported by other groups in that we find few overlapping lipid alterations between brain and serum (Arnold et al., 2020; Batra et al., 2022). We identified a similar lipid profile difference in DLPFC brain tissue in NCI+ and MCI− (Figure 3), as well as a biphasic difference in the mole percent of PC and LPC across disease progression (Figure 4; Supplementary Figure S6). Interestingly, the differential analysis of lipidomics in DLPFC brain tissue failed to identify significant differences between NCI and AD in both *APOEε4* carriers and non-carriers regardless of controlling for education (Figure 4; Supplementary Figures S6, S7). Synthesizing these findings indicates that the magnitude of the lipid profile shift is most differential early in disease state in those at higher risk for AD (i.e., *APOEε4* carriers with no cognitive impairment) or early in disease progression, in those with MCI.

In the serum analysis, most of the differential lipid species are acylcarnitines, which are differential primarily comparing NCI− and MCI+ groups. Interestingly, all significantly differential acylcarnitines are downregulated in the non-NCI− groups compared to the NCI− group in both DLPFC brain tissue and serum (Figure 1). Fatty acid metabolism is the exclusive source of medium and long chain acylcarnitines in contrast to short-chain acylcarnitines which are derived from fatty acid degradation, and metabolism of amino acids and glucose (Makrecka-Kuka et al., 2017; Melone et al., 2018; Li et al., 2019; Fernandez and Ellis, 2020). Therefore, since acylcarnitines are reflective of free fatty acid content, the product of PLA2 activity, this further implicates dysregulation of the Lands cycle because of uncoupling between the expected increase of FFA/acylcarnitine and the observed decrease which is concurrent with a decrease in PC species. Specifically, a greater PLA2 activity would lead to a higher free fatty acid content and, potentially, acylcarnitine content. However, we found that acylcarnitines were lower, which suggests a change in Lands cycle utilization of FFA as substrates for acyl-Co-A synthesis and the Lands cycle, as well as a change in brain energy utilization, as has been previously described in AD and MCI (Cunnane et al., 2011; Toledo et al., 2017; Tyrrell et al., 2017; Yu et al., 2022). From serum, the MCI+ diagnosis group shows enrichment in a specific cluster, unlike other groups (Figure 2). The red cluster is driven by expression of genes within the serum turquoise module, and more than half of all MCI+ samples are part of the red cluster, as the reviewer points out. From the turquoise module, the major components driving the grouping of the red cluster are C12 and C14.2, with module loadings 0.354 and 0.334, respectively. Across the six diagnosis groups, the median abundance levels of both of these lipids is at its lowest in the MCI+ group. For the remaining lipids in this module, we see a similar trend (of lower magnitude) of down regulation in the MCI+ enriched red cluster compared to the remaining clusters.

Our study identified a lipid profile in *APOEε4* carriers and MCI non-carriers, implicating dysregulation of the Lands cycle acyl chain remodeling (Supplementary Figure S9). However, based on the panel of lipids represented in the Biocrates p180 panel (Arnold et al., 2020), we cannot exclude other lipid pathways from dyshomeostasis early in disease or due to the *APOEε4* genotype. Future studies utilizing a lipidomic panel with increased coverage across lipid classes and species are critical for identifying a system wide understanding of lipid metabolism in AD brain. Importantly, cluster assignments differentiated NCI+ in the red cluster where a majority of MCI− samples are also present, supporting the similarity between *APOEε4* carriers (NCI+) and MCI non-carriers (MCI−) (Figures 3E,F). It is also important to note that the orange cluster was enriched in AD samples, indicating the dissimilar lipid profile shifts found in MCI and AD. This suggests non-linear, biphasic, changes in lipid profiles during disease progression from NCI to MCI to AD.

Our findings have shown that *APOEε4* carriers and MCI non-carriers have a similar lipid profile when the level of education is not taken into account. When education is considered as a covariate, the similarity in the lipid profiles is no longer strongly correlated (Supplementary Figure S7). Though the correlations are not significant, it is clear that *APOEε4* carriers have a higher eigen lipid value, a trend that is also seen in MCI non-carriers (MCI−) (Figure 5A), suggesting that the same lipid profile is present in *APOEε4* carriers, who are highly educated in this cohort, and MCI non-carriers. Interestingly, the *APOEε4* carriers at the stage of MCI (MCI+) are very sensitive to differences in the eigen lipid profile, which are correlated with higher CGRS, suggesting improved cognition with higher levels of the eigen lipids in the blue module (Figure 5B).

In the ROSMAP subgroup analyzed for lipidomics in this study, the education level of the *APOEε4* carriers is the highest of all the groups. In previous studies, *APOEε4* has been associated with worse cognition and earlier death (Robinson et al., 2020; Gharbi-Meliani et al., 2021; Pavel et al., 2022); therefore, our study may indicate that these cognitively normal *APOEε4* carriers in this study are resilient and display high cognitive reserve. Further, the age of death in this subset is higher than expected for *APOEε4* carriers (Robinson et al., 2020; Pavel et al., 2022), which may represent selection bias for *APOEε4* carriers in the subset of this postmortem study and not general *APOEε4* attributes. The *APOEε4* carriers (*n* = 6) in this study who are not cognitively impaired (NCI+) have higher education levels than the other groups, potentially through selection bias in the ROSMAP cohort, and thus may have maintained their non-impaired status (despite higher risk) through putative mechanisms of resilience (Table 1). This suggests that the lipid profile we have shown indicates a resilience profile that is present in MCI non-carriers and lost in later stage AD. Thus, lipid differences in brain tissue from MCI non-carriers may be compensatory to an initial challenge to lipid metabolism, which is likely to target acyl chain remodeling in both carriers and non-carriers of the *APOEε4* allele at different stages of disease. However, the number (*N*) of *APOEε4* carriers at all stages of the disease is small (Table 1) and will need to be increased to fully ascertain the effect of carriership on lipid content of brain and serum.

Ultimately, the ability to determine clinical progression is paramount to identification of biomarkers, so to determine if lipids were associated with cognitive decline, we used multiple linear models to examine the associations between specific lipid species and a relevant measure for clinical AD—cognitive global random slope (CGRS). Within these models, we controlled for age, sex and education in both brain and serum (Figure 5). We discovered two lipids (both PCs), which were identified in the blue module of the brain data, that were positively associated with cognitive function: PC.aa.C36.0 and PC.ae.C38.1 (Figures 3D, 5C,D), suggesting a protective role. Importantly, when we controlled for amyloid plaque and tau tangle pathologies using a pathological measures-adjusted version of CGRS, these lipids did not remain significantly associated with CGRS in contrast to the genes involved in phospholipid metabolism. Further studies will be required to understand the mechanistic role of these lipid species in association with pathology and cognitive decline in the brain.

While our study has provided novel insights into differential lipidomic profiles in ApoEe4 carriers and at different stages of AD, it is important to acknowledge several limitations. Our reported findings are observational, and the cross-sectional design of the studies involving post-mortem tissue inherently restricts our ability to ascertain causal relationships. Consequently, we cannot definitively determine whether the observed changes in brain tissue directly contribute to disease development or if they are instead a consequence of the pathological alterations within the brain. Secondly, the heterogeneity estimates presented in this study require replication in an independent cohort with adequate sample sizes for meaningful stratification alongside the availability of lipidomics and endophenotypic data. Further, there is a bias for females in the *APOEε4* positive MCI and AD groups, and males are not well represented. Additional studies are necessary to determine if these findings are dependent on sex, as a has been shown previously in the serum metabolome (Arnold et al., 2020). Finally, the p180 Biocrates panel is not comprehensive and represents a limited lipid species and class profile. Future studies will aim to acquire a full lipidomic profile in plasma and post-mortem brain tissue, including additional anatomical regions, to determine the extent of acyl chain remodeling deficits across multiple phospholipid classes as well as potential identification of other dysregulated pathways in lipid metabolism.

Our study identifies a common lipid profile shift between non-cognitively impaired *APOEε4* carriers who are highly educated in this cohort and may represent a resilient population and MCI *APOEε4* non-carriers, supporting a common disease etiology based on lipid content in DLPFC brain tissue and supports a non-linear, biphasic, shift in the lipid profile across disease progression.

## Data availability statement

The data analyzed in this study is subject to the following licenses/restrictions: Data Use Agreement. Requests to access these datasets should be directed to https://www.synapse.org/#!Synapse:syn26007829; https://www.synapse.org/#!Synapse:syn3388564. All code supporting the findings of this study are available to qualified individuals upon request. Please contact LM (lbm7002@med.cornell.edu) for access.

## Ethics statement

The studies involving humans were approved by Institutional Review Board of Rush University Medical Center. The studies were conducted in accordance with the local legislation and institutional requirements. Written informed consent for participation was not required from the participants or the participants’ legal guardians/next of kin in accordance with the national legislation and institutional requirements.

## Author contributions

JM: Conceptualization, Data curation, Formal analysis, Methodology, Validation, Visualization, Writing – original draft, Writing – review & editing. AC: Conceptualization, Investigation, Methodology, Validation, Visualization, Writing – original draft, Writing – review & editing. WD: Investigation, Methodology, Validation, Visualization, Writing – original draft, Writing – review & editing. KW: Visualization, Writing – review & editing. AL: Writing – review & editing. DB: Conceptualization, Data curation, Funding acquisition, Methodology, Project administration, Resources, Supervision, Writing – review & editing. TN: Conceptualization, Validation, Writing – original draft, Writing – review & editing, Investigation. VM: Conceptualization, Formal analysis, Investigation, Methodology, Project administration, Supervision, Validation, Visualization, Writing – original draft, Writing – review & editing. LM: Conceptualization, Funding acquisition, Investigation, Methodology, Project administration, Supervision, Validation, Visualization, Writing – original draft, Writing – review & editing.
