# Corrigendum: Brain and serum lipidomic profiles implicate Lands cycle acyl chain remodeling association with *APOE*ε*4* and mild cognitive impairment

**DOI:** 10.3389/fnagi.2024.1480818

**Published:** 2024-09-25

**Authors:** Jason Mares, Ana Paula Costa, William J. Dartora, Krista M. Wartchow, Artur Lazarian, David A. Bennett, Tal Nuriel, Vilas Menon, Laura Beth J. McIntire

**Affiliations:** ^1^Center for Translational & Computational Neuroimmunology, Department of Neurology, Taub Institute for Research on Alzheimer's Disease and the Aging Brain, Columbia University Irving Medical Center, New York, NY, United States; ^2^Lipidomics and Biomarker Discovery Lab, Department of Radiology, Brain Health Imaging Institute, Weill Cornell Medicine, New York, NY, United States; ^3^Rush Alzheimer's Disease Center, Rush University Medical Center, Chicago, IL, United States; ^4^Department of Pathology and Cell Biology, Taub Institute for Research on Alzheimer's Disease and the Aging Brain, Columbia University Irving Medical Center, New York, NY, United States

**Keywords:** lipidomics, lipid metabolism, Alzheimer's disease, ROSMAP, ApoE

In the published article, there was an error in the caption for **Figure 5. Impact of lipid species in the dorsolateral prefrontal cortex and serum on cognition controlling for educational background and neuropathological markers** as published. The caption text in Figure 5 was incorrectly stated as: **(A)** Multiple regression models for cognitive global random slope as the dependent variable are conducted for brain and **(B)** serum. Volcano plots present the main results of the regressions with lipid species coefficient and BH-adjusted *p*-values along the *x* and *y* axes, respectively. **(C)** Models are re-run accounting for amyloid plaques and tau tangles markers in brain and **(D)** serum.

The corrected caption appears below.

**Figure 5. Impact of lipid species in the dorsolateral prefrontal cortex and serum on cognition controlling for educational background and neuropathological markers**. **(A)** Education-adjusted scores for the blue eigen module are shown in diagnosis-genotype groups. **(B)** Association of the residuals of education-adjusted blue modules with cognitive global random slope are shown and post-hoc linear regression lines are added onto the plot. The left-top table reports correlation test results between residuals and cognitive global random slope for each diagnosis-genotype group. **(C, D)** Using linear models, we analyzed the association of the cognitive global random slope with each lipid species in the **(C)** DLPFC and **(D)** serum data. Volcano plots present the main results of these models with lipid species beta estimates and Benjamini-Hochberg-adjusted *p*-values shown along the *x* and *y* axes, respectively and significant results are shown above the threshold (dashed line).

The authors apologize for this error and state that this does not change the scientific conclusions of the article in any way. The original article has been updated.

