# Supplementary figures and images for: Brain and serum lipidomic profiles implicate Lands cycle acyl chain remodeling association with APOEε4 and mild cognitive impairment
Source: Front Aging Neurosci. 2024 Jun 11;16:1419253. doi: 10.3389/fnagi.2024.1419253 (PMC11210445; doi:10.3389/fnagi.2024.1419253)

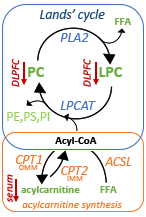

Supplement: Supplementary file 9 [file Image_9.TIF]
